# Deep Brain Stimulation-Induced Transient Effects in the Habenula

**DOI:** 10.3389/fpsyt.2021.674962

**Published:** 2021-06-23

**Authors:** Chencheng Zhang, Yijie Lai, Yingying Zhang, Xinmeng Xu, Bomin Sun, Dianyou Li

**Affiliations:** ^1^Clinical Neuroscience Center, Ruijin Hospital Luwan Branch, Shanghai Jiao Tong University School of Medicine, Shanghai, China; ^2^Department of Neurosurgery, Center for Functional Neurosurgery, Ruijin Hospital, Shanghai Jiao Tong University School of Medicine, Shanghai, China

**Keywords:** deep brain stimulation, habenula, acute electrical stimulation, bipolar disorder, schizophrenia

## Abstract

The habenula, located in the epithalamus, has been implicated in various psychiatric disorders including mood disorders and schizophrenia. This study explored the transient effects of deep brain stimulation in the habenula. Each of the four patients (two with bipolar disorder and two with schizophrenia) was tested with eight deep brain stimulation contacts. Patients were examined via transient electrical stimulation 1 month after deep brain stimulation surgery. The pulse width was 60 μs and the voltage ranged from 0 V to a maximum of 10 V, increasing in increments of 1 V. Each patient received stimulation at two frequencies, 60 and 135 Hz. A total of 221 out of 385 active trials elicited stimulation-induced effects. The three most common transient effects were numbness, heart rate changes, and pain. The incidence of numbness, heart rate changes, pain, and involuntary movements increased with the increase in stimulation voltage. Through contralateral stimulation, numbness was triggered in all parts of the body except the scalp. The obtained stimulus-response maps suggested a possible somatosensory organization of the habenula.

## Introduction

The habenula is a paired, evolutionarily conserved structure located in the epithalamus. It connects more recently evolved structures in the neocortex and limbic forebrain that are responsible for executive functions with ancient areas in the midbrain and hindbrain that process sleep, pain, and reward (1). Many preclinical studies have acknowledged this structure in psychiatric disorders such as mood disorders and schizophrenia (2).

Deep brain stimulation (DBS) involves the implantation of electrodes within specific areas of the brain and the generation of electrical impulses that control abnormal brain activity. The amount of stimulation in DBS is controlled by a pacemaker-like device which is usually placed under the skin in the upper chest. A wire traveling beneath the skin connects the device to the electrodes in the brain. DBS has been approved for the treatment of various conditions including dystonia, epilepsy, essential tremor, obsessive-compulsive disorder, and Parkinson's disease (3). DBS is also investigated as a potential treatment for addiction (4), major depression (5), and Tourette syndrome (6). Physicians may use DBS to treat movement disorders or neuropsychiatric disorders if medications become less effective or if poorly tolerated. Unlike other surgical options, an advantage of DBS is that it is reversible and does not cause permanent damage to any part of the brain (7). Therefore, deep brain stimulation is seen as a promising treatment for psychiatric disorders. In two clinical trials targeting the habenula (5, 8), we systematically assessed transient responses to focal electrical stimulation.

## Methods

**Figure 1:**
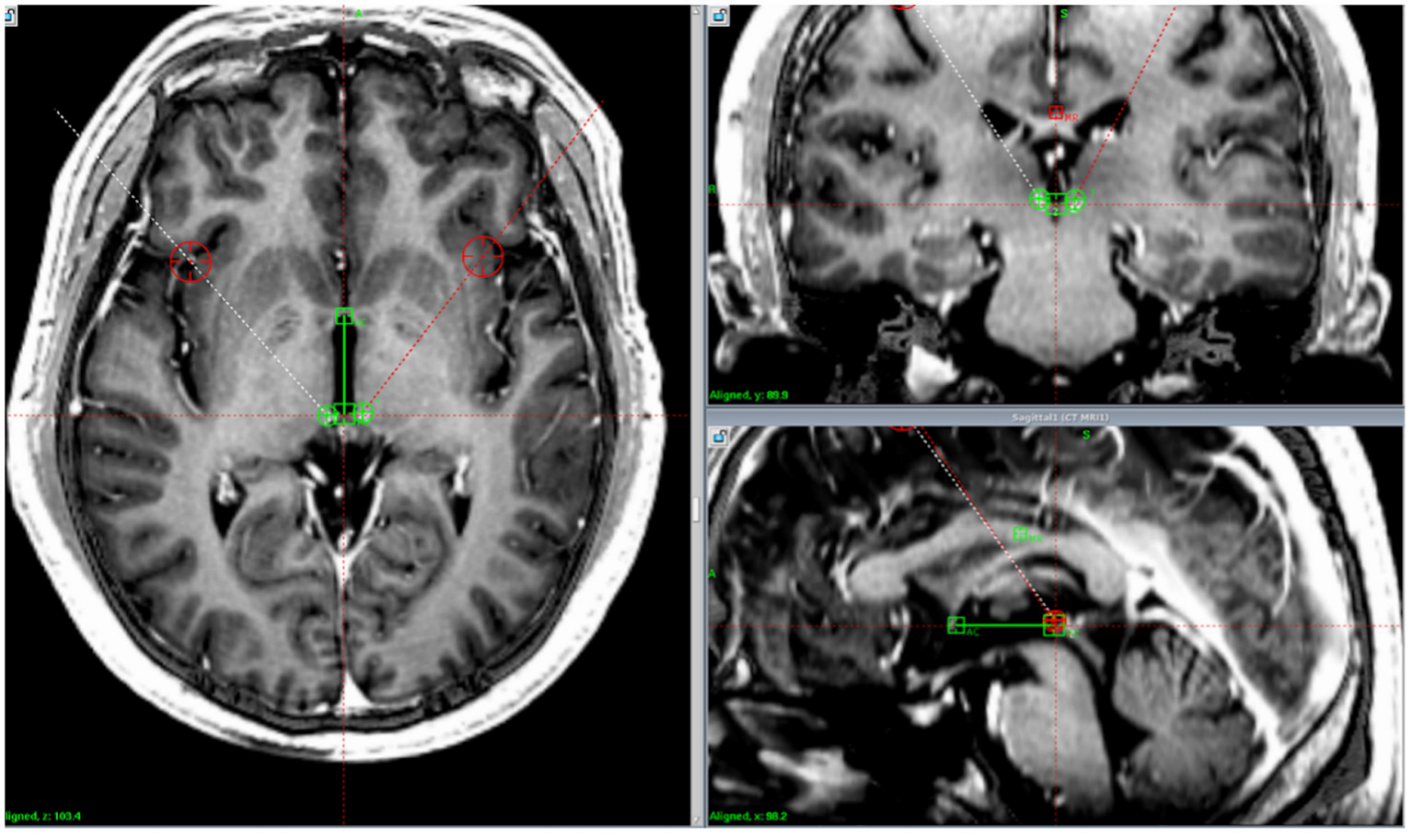
Lead location. Postoperative computed tomography images fused with preoperative magnetic resonance imaging demonstrating the position of the implanted electrodes in the habenula in one patient.

**Figure 2:**
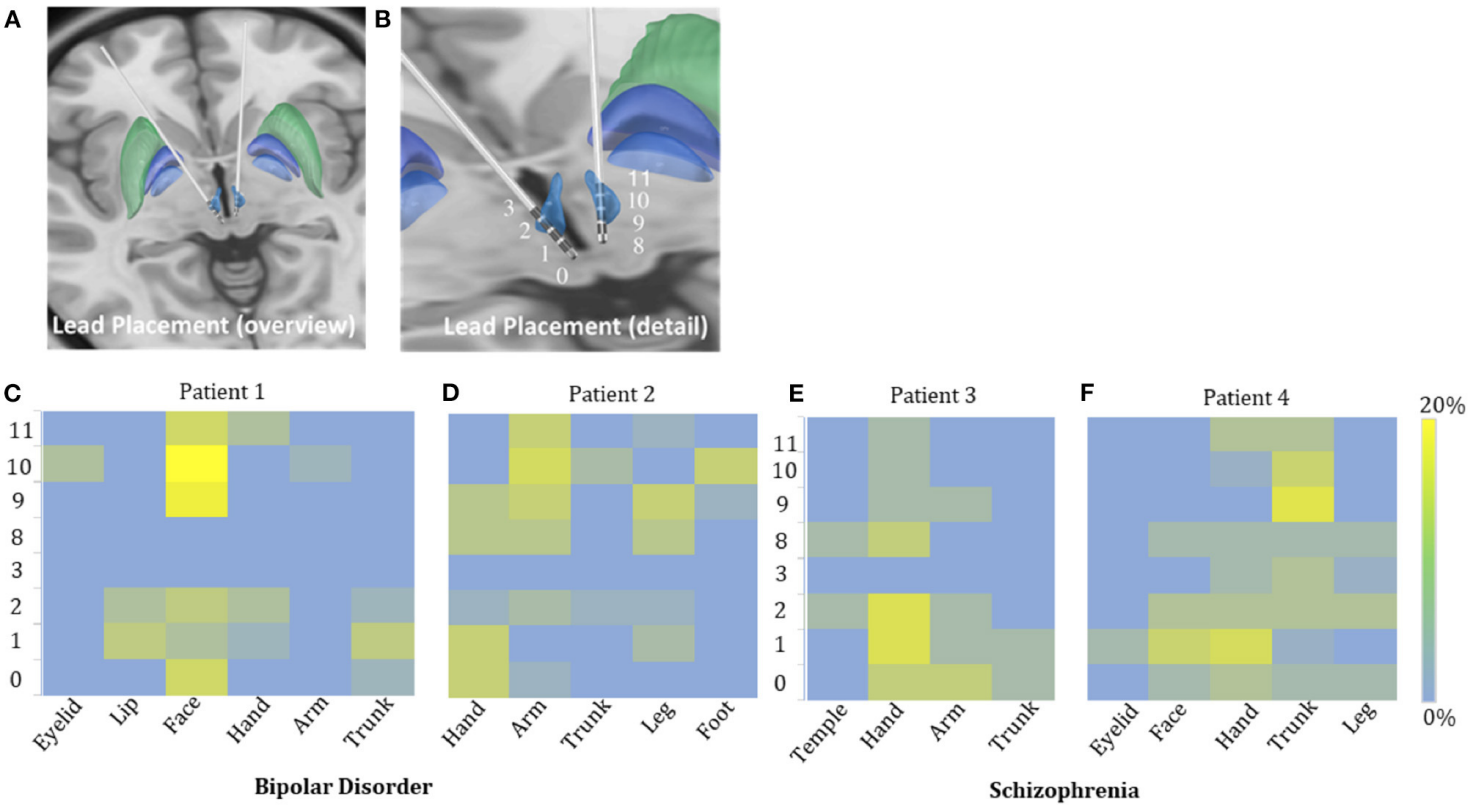
Possible sensory projections in the habenula. Overview **(A)** and detailed view **(B)** of the deep brain stimulation lead placement. The letters marked beside the cathodes **(C–F)** indicate the most frequently reported body part affected by numbness. The colors box indicated the percentage of numbness occurrence at different contact and body parts in numbness trials for each patient. The contacts 0, 1, 2, and 3 were located in the right habenula from ventral to dorsal, and the contacts 8, 9, 10, and 11 were located in the left habenula from ventral to dorsal.

Four patients with refractory psychiatric disorders [Table 1, two with schizophrenia (8) and two with bipolar disorder (5)] underwent DBS placement in the bilateral habenula (Figures 1, 2A,B). All the patients were male with a mean age of 31 years. The test was conducted at 1 month after surgery. The stimulation pulse width was fixed at 60 μs and the voltage was systematically increased from 0 V to a maximum of 10 V in increments of 1 V. Our test stimulation was delivered unilaterally on each contact in each patient. The contact was tested from ventral to dorsal areas and the frequency was set at low frequency (60 Hz) and titrated to high frequency (135 Hz) later in the test. If the patient reported an unacceptable side-effect, trials with higher voltage amplitudes at that contact were waived. Meanwhile, expecting that 0 V stimulation (no stimulation) would cause little effects on the patients, we decided to reduce the number of sham trials to facilitate the progression of the experiment. Patients underwent single blind tests during which they sat in a chair facing a video camera and their heart rates were recorded. A programmer sat behind the patient and a recorder sat behind the camera. The programmer used gestures to inform the recorder of the voltage used. At the beginning of each trial, parameters of DBS were changed, and the patient was asked to describe what they felt in a time window of 2 min. The association between stimulation parameters and different stimulation-induced effects was examined using logistic regression and a two tailed *p* < 0.05 was considered significant. Positions of the electrodes in the nucleus were reconstructed using the Lead-DBS toolbox in MATLAB according to the methods described by Horn et al. (9). For each patient, we also explored the affected body parts in numbness trials. All participants provided written consent and the experimental protocol was approved by the ethics committee of Ruijin Hospital, Shanghai Jiao Tong University School of Medicine.

**Table 1:** Conditions of the different trials.

**Variables**	**Specific conditions**	**Trials**	**Proportion (%)**
Frequency (Hz)	60	195	50.6%
	135	190	49.4%
Diagnosis	BP	211	54.8%
	SZ	174	45.2%
Voltage (V)	1	25	6.5%
	2	58	15.1%
	3	38	9.9%
	4	60	15.6%
	5	41	10.6%
	6	56	14.5%
	7	26	6.8%
	8	39	10.1%
	9	18	4.7%
	10	24	6.2%
Patient	NO. 1	127	33.0%
	NO. 2	84	21.8%
	NO. 3	91	23.6%
	NO. 4	83	21.6%
Cathode	0	42	10.9%
	1	51	13.2%
	2	49	12.7%
	3	47	12.2%
	8	42	10.9%
	9	51	13.2%
	10	51	13.2%
	11	52	13.5%

*BP, bipolar disorder; SZ, schizophrenia*.

## Results

A total of 385 active and 9 sham trials were tested (Table 2); 219 active and no sham trials induced transient stimulation-induced effects. The most common responses were numbness (66.7% of 219 trials), heart rate changes (36.1%), pain (16.9%) and dizziness (16.0%). Other transient effects included eye closure (11.0%), involuntary movements (8.2%), giddiness (8.2%), chest pain (6.4%), nausea (5.5%), discomfort (3.2%), a feeling of relaxation (2.3%), and a feeling of heaviness in some body parts (0.9%). One patient with bipolar disorder also reported in some trials a feeling of electrical shock (5.9%) and palpitation (1.8%) (Table 3). No severe adverse events were observed.

**Table 2:** Relationships of trial conditions with responses.

**Response**	**Number of responses**	**Prop. of responses (%)**	**Lead contact**	**Voltage**	**Frequency**	**Diagnosis**	**Patient**
Numbness	146	66.7	0.197	0.015^*^	0.702	0.848	<0.0001^***^
Changes in heart rate	79	36.1	0.635	0.071	<0.0001^***^	<0.0001^***^	<0.0001^***^
Pain	37^a^	16.9	0.573	0.425	0.454	<0.0001^***^	<0.0001^***^
Dizziness	35	16	0.002^**^	0.001^**^	0.003^**^	0.447	0.001^**^
Eye closure	24^b^	11	0.595	0.335	0.157	0.058	<0.0001^***^
Giddiness	18	8.2	0.036^*^	<0.0001^***^	<0.0001^***^	0.001^**^	<0.0001^***^
Involuntary movements	18^c^	8.2	0.008^**^	0.534	0.553	0.001^**^	<0.0001^***^

*Responses that occurred in <5% of the trials or in only one patient were omitted. Prop, proportion*.

* *p < 0.05;*

** *p < 0.01;*

*** *p < 0.001*.

*The total number of trials was 219*.

a *36 reported in a patient with schizophrenia;*

b *15 reported in another patient with schizophrenia;*

c *12 reported in a patient with bipolar disorder*.

**Table 3:** Demographic and clinical information.

**Patient**	**NO.1**	**NO.2**	**NO.3**	**NO.4**
Diagnosis	Bipolar disorder I	Bipolar disorder I	Schizophrenia	Schizophrenia
Comorbidity	No	Substance dependence (clonazepam, alcohol gambling disorder)	No	No
Age at surgery	41	46	26	21
Gender	Male	Male	Male	Male
Education (years)	12	9	10	10
Marriage	Married	Divorced	Single	Single
Duration of disease (years)	21	10	9	4
Current episode duration (years)	3.5	10	9	4
Past ETC	Yes	Yes	Yes	Yes
Medications per day	Clomipramine hydrochloride,150 m; Ozanpine, 5 mg	Lamotrigine, 100 mg; seroquel, 100 mg; magnesium valproate, 0.75 g; amfebutamone, 0.3 g; clonazepam, 16–20 mg	Ozanpine,10 mg; lithium carbonate, 0.25 g	Quetiapine, 0.2 g; benzhexol, 4 mg; ziprasidone hydrochloride, 60 mg
HAMD	24	23	NA	NA
YMRS	0	11	NA	NA
PANSS	NA	NA	74	66
Positive subscale	NA	NA	13	14
Negative subscale	NA	NA	23	19
General subscale	NA	NA	38	33

*HAMD, Hamilton depression rating scale; YMRS, Yong mania rating scale; PANSS, Positive and negative syndrome scale; ECT, Electroconvulsive therapy; NA, Not available*.

The incidence of numbness (*p* = 0.015), heart rate change (*p* = 0.071), pain (*p* = 0.425), and involuntary movements (*p* = 0.534) increased as the DBS voltage increased. It appeared more often for the 60-Hz voltage pulse to evoke an increase in heart rate (*p* < 0.0001) and less often to induce dizziness than for the 135-Hz voltage pulse (*p* = 0.003). DBS elicited heart rate change (*p* < 0.0001) and pain (*p* < 0.0001) more often in patients with schizophrenia than with bipolar disorder. On the other hand, patients with bipolar disorder exhibited giddiness (*p* = 0.001) and involuntary movements (*p* = 0.001) more often. Specifically, under active stimulation, one patient with schizophrenia reported feelings of pain in almost all trials (36 out of 37 = 97.3%); the other patient with schizophrenia experienced eye closure in more than half of the trials (15 out of 24 = 62.5%); one patient with bipolar disorder reported involuntary movements in 12 out of 18 trials (66.7%). The experience of numbness except on scalp was predominantly triggered by contralateral stimulation. The most frequently affected body parts in these four patients were face (26 out of 35 = 74.3%), arm (19 out of 41 = 46.3%), hand (13 out of 22 = 59.1%) and trunk (26 out of 48 = 54.2%), respectively (Figures 2C–F).

## Discussion

### Somatosensory Organization of the Habenula

The safety and feasibility of DBS in the habenula for various refractory psychiatric disorders have been demonstrated in previous studies. In this study, we observed a lateralized pattern of stimulation-induced responses: the left side of the habenula appeared to correlate only with sensations in the right arm, while the right side of the habenula correlated with sensations in the left face, leg, and hand. Subject variability in the location of the active contact of each electrode could be a confounding factor. Unfortunately, the post-surgical imaging was not helpful in clarifying this issue.

### Various Effects Induced by Different Combination of Stimulation Parameters

Parameter settings have been discussed extensively in terms of movement disorders. For instance, 130 Hz is used as a standard frequency for DBS in Parkinson's disease because it balances between power consumption and clinical efficiency. A different choice of frequency may be applied to treat mood disorders, especially when the electrodes are placed at newly discovered brain targets. Low frequency or high frequency DBS in the habenula has been suggested to induce physiological effects relevant to habenula functions such as mood regulation in bipolar disorders. The patients' reactions to the two tested frequencies (60 and 135 Hz), namely changes in heart rate and feelings of dizziness, were significantly different, suggesting that the lower stimulation frequency was more likely to affect the heart rate while the higher frequency was more likely to affect sensations of dizziness.

### Presumed Mechanisms Behind the Various Transient Effects

DBS is used to treat neuropsychiatric conditions by providing certain electrical stimulation to certain brain regions. However, DBS affects not just the targeted area but also other components in the neural network. The lateral habenula is involved in pain transmission by receiving pain signals from the spinal cord and interacting with canonical pain modulatory regions. The lateral habenula is also connected with hypothalamus which is known as an autonomic regulatory region (2). Therefore, the heart rate changes may be induced by the regulation of the central autonomic regulatory regions from the habenula.

### Limitations

First, only four patients with psychiatric disorders were tested: the small sample size might limit the validity and generality of the study. Second, our results were based mainly on the patients' subjective descriptions which potentially introduced self-report bias. Also, specific psychiatric responses (i.e., mood, psychosis, and anxiety) were not monitored during testing.

To our knowledge, this is the first study to investigate transient effects induced by systematic electrical stimulation of the habenula. We have demonstrated the effect profile and have proposed a possible somatosensory organization in the habenula. The most common transient effect was numbness, followed by heart rate changes and pain. Different frequencies appeared to elicit similar responses in most cases. The relationship between transient effects and long-term clinical outcomes requires further investigation.

## Data Availability Statement

The raw data supporting the conclusions of this article will be made available by the authors, without undue reservation.

## Ethics Statement

The studies involving human participants were reviewed and approved by Ruijin Hospital. The patients/participants provided their written informed consent to participate in this study.

## Author Contributions

DL and BS designed the study. DL conducted the testing. YZ collected the data. YL analyzed the data. CZ wrote the manuscript. YZ and YL drafted the figures. XX reviewed and revised the manuscript and wrote the abstract. All authors contributed to the article and approved the submitted version.

## Conflict of Interest

The authors declare that the research was conducted in the absence of any commercial or financial relationships that could be construed as a potential conflict of interest.
